# Development and application of a real-time VPCR method for ultra-fast quantitative analysis and Visual Detection of HBV-DNA

**DOI:** 10.1128/jcm.00154-26

**Published:** 2026-08-14

**Authors:** Tingting Zhao, Tongyi Peng, Jiaqi Cui, Yan Chen, Wenjun Song, Hongling Chen, Bo Wu, Rong Chen

**Affiliations:** 1 School of pharmacy, School of Ethnic Medicine, Chinese Medicine Germplasm Resources Innovation and Effective Uses Key Laboratory of Sichuan Province, Chengdu University of Traditional Chinese Medicine, Chengdu, Sichuan, China; 2 Department of Clinical Laboratory, Hospital of Chengdu University of Traditional Chinese Medicine, Chengdu, Sichuan, China; Mayo Clinic Minnesota, Rochester, Minnesota, USA

**Keywords:** hepatitis B virus infection, real-time VPCR, quantitative detection system, fluorescence visual detection

## Abstract

Hepatitis B virus (HBV) infection remains a major global health challenge, necessitating rapid and accurate diagnostic methods for effective disease management. Although quantitative real-time PCR (qPCR) is widely used, its detection time is relatively long. We developed an ultra-fast HBV-DNA quantification system based on rapid VPCR technology, which, combined with a 10-min nucleic acid extraction process, reduces the entire workflow to approximately 35 min. Crucially, despite this significant increase in speed, the system maintains analytical sensitivity, specificity, and reproducibility comparable to those of conventional qPCR. The system’s performance was then evaluated using 143 clinical samples and compared to that of standard qPCR, demonstrating that the developed real-time VPCR system significantly shortened the amplification time while maintaining excellent performance, with 100% sensitivity and 97.09% specificity. Additionally, a cost-effective qualitative endpoint visual detection method based on probe cleavage was established, offering an alternative readout strategy suitable for HBV screening in resource-limited settings without requiring expensive instruments. Collectively, this study presents a rapid quantification system and a qualitative visual detection method that significantly enhance diagnostic efficiency, providing strong technical support for clinical HBV management and screening.

**IMPORTANCE:**

The rapid hepatitis B virus (HBV) quantification system established in this study completes the entire workflow within 35 min and delivers excellent performance, meeting the demand for rapid decision-making in time-sensitive scenarios such as emergency care and acute liver failure. Its “test-and-treat” workflow is particularly suitable for patients in remote areas, allowing diagnosis and treatment in a single visit. Meanwhile, the accompanying visual qualitative method provides a low-cost screening strategy for resource-limited settings. This system greatly improves detection efficiency and clinical accessibility, showing important application value for the rapid diagnosis of hepatitis B and other pathogens.

## INTRODUCTION

Hepatitis B virus (HBV) infection is a global health issue and a leading cause of chronic liver disease. It is estimated that 257 to 292 million people worldwide are infected with HBV, with over 95% of these patients living in low- and middle-income countries. However, only 12% to 25% of these patients have access to diagnosis and antiviral treatment (1, 2). HBV is a hepatotropic DNA virus primarily transmitted through blood, mother-to-child transmission (3), and medical procedures. Once it invades the human body, it can lead to cellular immune evasion, causing a series of continuous liver inflammation responses that may eventually progress to cirrhosis or even liver cancer (4). Despite the development of vaccines against hepatitis B and other disease control measures, including antiviral treatments, the management of hepatitis B remains a challenging issue (5, 6). Research has shown that timely detection of HBV replication, along with prompt treatment, can effectively control disease progression and significantly improve patient prognosis (7–9). In addition, studies have shown that low- and middle-income countries lack HBV testing, have low awareness of the disease, and face high testing costs (10, 11). As such, the World Health Organization (WHO) has implemented actions and set goals aimed at eliminating viral hepatitis by 2030, with one of the main objectives being to improve the efficiency and coverage of HBV testing (12, 13). Consequently, the establishment of rapid, simple, and cost-effective HBV detection methods is crucial for the diagnosis and treatment of HBV (14).

**Fig 1:**
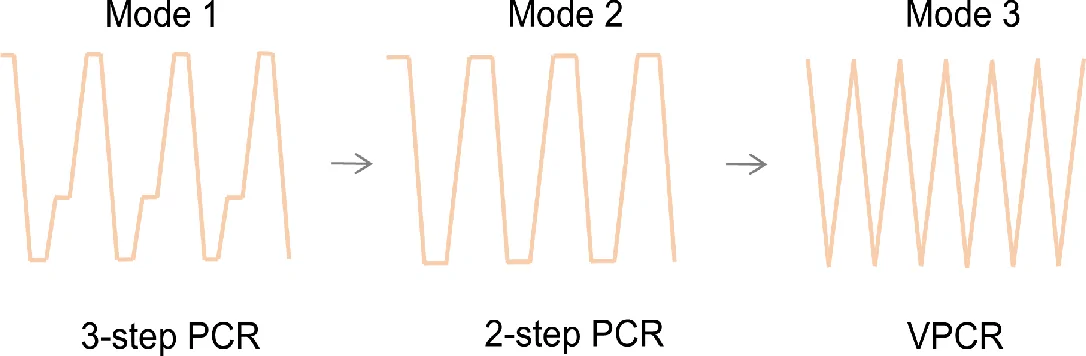
Temperature-time curve of the three-step PCR, two-step PCR, and VPCR methods. Reproduced with permission from reference **20**.

Currently available methods for detecting HBV DNA include quantitative real-time PCR (15), recombinase polymerase amplification (RPA) (16), loop-mediated isothermal amplification (LAMP) (17), and CRISPR/Cas technology (18). Among these, qPCR is the most widely used in clinical settings, typically requiring 2–3 h to complete the entire process (19). Previous studies by our research group have shown that PCR amplification need not strictly conform to the traditional three-step or two-step protocols with fixed temperature cycles. Instead, the process can be completed during a dynamic temperature ramping phase, which significantly shortens the overall reaction time. Because the temperature-time profile of this amplification system displays multiple “V”-shaped patterns, the method has been named “VPCR.” Importantly, while retaining the analytical sensitivity and specificity offered by existing instruments, this method reduces the nucleic acid amplification time to approximately one-third of that required by traditional methods (20–24) (Fig. 1). To date, this technology has been adopted by multiple research laboratories for academic studies (25–28). This study aims to develop a rapid VPCR quantitative detection system for HBV DNA based on the amplification principle of VPCR technology. By utilizing the existing instruments, the system enables the rapid detection of HBV DNA. This provides a scientifically effective technical solution to shorten clinical detection time and improve detection efficiency.

## MATERIALS AND METHODS

The hepatitis B virus (HBV) nucleic acid detection kit (PCR-fluorescent probe method) was purchased from Guangzhou Da’an Gene Co., Ltd. The hepatitis A virus (HAV), hepatitis C virus (HCV), hepatitis D virus (HDV), hepatitis E virus (HEV), human immunodeficiency virus (HIV), herpes simplex virus type 1 (HSV-1), and pseudoviruses carrying cowpox virus and monkeypox virus DNA were kindly provided by the Chengdu Institute of Biology, Chinese Academy of Sciences. The DNA of 13 respiratory pathogens, namely, influenza A virus (IAV) (H7N9, H1N1, H3N2, and H5N2), influenza A virus H1N1 (IAV-H1N1) (2009 pandemic strain), seasonal influenza A virus H3N2 (IAV-H3N2), influenza B virus (IBV) (Victoria lineage and Yamagata lineage), adenovirus (AdV) (Groups B, C, and E), human bocavirus (HBoV), human rhinovirus (HRV), parainfluenza virus (PIV) (types 1, 2, 3, and 4), human coronavirus (HCoV) (229E, OC43, NL63, and HKU1), respiratory syncytial virus (RSV) (Groups A and B), human metapneumovirus (HMPV), *Mycoplasma pneumoniae* (Mp), and Chlamydia (*Chlamydia trachomatis* [CT] and *Chlamydia pneumoniae* [CPn]), was obtained from Ningbo Health Gene Technologies Co., Ltd. Additionally, *Staphylococcus aureus*, *Pseudomonas aeruginosa*, *Escherichia coli*, *Salmonella typhi*, *Staphylococcus epidermidis*, and *Listeria* were provided by the Chengdu Food Inspection Research Institute.

### HBV standard serum and clinical samples

HBV standard serum, with concentrations ranging from 2 × 10^5^ to 2 × 10^1^ IU/mL, was obtained from an HBV nucleic acid detection kit (PCR fluorescence probe method) manufactured by Guangzhou Da’an Gene Co., Ltd.

A total of 191 qualified residual clinical specimens were obtained from the Department of Laboratory Medicine, Affiliated Hospital of Chengdu University of Traditional Chinese Medicine. All specimens were collected from patients with a known history of HBV infection who required viral load monitoring during treatment.

### Sample processing

Clinical samples were collected as peripheral venous blood. For each sample, 2 mL of whole blood was centrifuged at 1,500 rpm for 5 min to separate serum, which was then used for downstream DNA extraction. HBV standard serum was processed directly for DNA extraction. Viral DNA was extracted using the FastPure Viral DNA/RNA Mini Kit V2 (Vazyme) in strict accordance with the manufacturer’s instructions, and the entire extraction process took approximately 10 min.

### Design of primers and probes and preparation of plasmids

HBV-specific primers and probe were designed based on the S region sequence of hepatitis B virus and validated using the Basic Local Alignment Search Tool (BLAST) tool provided by the National Center for Biotechnology Information (NCBI). RNAse P was selected as the internal control gene. The primers and probe used for RNAse P amplification were based on the protocol described in reference 29. All primers and probes used in this study were synthesized and purified by Sangon Biotech (Shanghai) Co., Ltd. The sequences of the primers and probes are listed in Table 1.

**TABLE 1:** Primers and probe sequence information

Name	Sequence (5′–3′)	Bases
DA-F	CTGTTCAAGCCTCCAAGCTGT	21
DA-R	GTCAGAAGGCAAAAAAGAGAGTAACTCC	28
DA-P	FAM-CCTTGGGTGGCTTTAGGGCATGGACATTGA-BHQ1	30
Rnase-fp	AGATTTGGACCTGCGAGCG	19
Rnase-rp	GAGCGGCTGTCTCCACAAGT	20
Rnase-p	VIC-TTCTGACCTGAAGGCTCTGCGCG-BHQ1	23

### Reaction mixture and cycling conditions for real-time VPCR and conventional quantitative real-time PCR (qPCR)

The total volume of the real-time VPCR reaction system is 25 μL, consisting of 6 mM Mg^2+^ (concentration 100 mmol/L), 2.5 μL of 10 × Easy Taq Buffer, 0.5 μL of Easy Taq enzyme, 0.5 μL of dNTPs (concentration 10 mmol/L), 10 μL of the HBV-DNA template, 2.5 μL of DA-F (concentration 10 mmol/L), 2.5 μL of DA-R (concentration 10 mmol/L), 2.5 μL of DA-P (concentration 10 mmol/L), 1.0 μL of Rnase-fp (concentration 10 mmol/L), 1.0 μL of Rnase-rp (concentration 10 mmol/L), 0.5 μL of Rnase-P (concentration 10 mmol/L), and ddH_2_O. The real-time VPCR program was conducted as follows: pre-denaturation at 93°C for 2 min; denaturation at 93°C for 0 s, annealing at 55°C for 0 s, 40 cycles, and total reaction time 25 min. Conventional qPCR assay, including the reaction setup and thermal cycling conditions, was performed strictly according to the manufacturer’s instructions for the Hepatitis B Virus Nucleic Acid Detection Kit, with a total amplification time of approximately 72 min (Daan Gene Co., Ltd.). All reactions were conducted on a Gentier 48E/48R real-time PCR instrument (Tianlong Technology, China).

### Assessment of analytical sensitivity and limit of detection

For analytical sensitivity evaluation, real-time VPCR and conventional quantitative real-time PCR(qPCR) were performed in parallel using DNA extracts prepared from HBV standard serum across a concentration range of 2 × 10^5^–2 × 10^1^ IU/mL. Ct values were recorded for all reactions, and the total run time was documented for each method. Standard curves were generated for each method by plotting Ct values against the log_10_ of the original concentration (IU/mL). Linearity was evaluated by linear regression analysis, and R^2^ was calculated to assess quantitative performance.

For limit of detection (LOD) evaluation, viral DNA was extracted from HBV standard serum serially diluted to 200, 20, 15, and 10 IU/mL following the DNA extraction procedure described in the sample processing section using the FastPure Viral DNA/RNA Mini Kit V2 (Vazyme). Each concentration was tested in 20 replicate reactions using the established real-time VPCR assay under identical reaction setup and thermal cycling conditions. The LOD was defined as the lowest concentration with a detection rate of at least 95% across the 20 replicates.

### Assessment of analytical specificity

For analytical specificity assessment, potential cross-reactivity was evaluated by testing a panel of non-target organisms as interference controls, including nucleic acids from hepatitis A virus, hepatitis C virus, hepatitis D virus, hepatitis E virus, human immunodeficiency virus, and herpes simplex virus 1, as well as DNA from variola virus and monkeypox virus. In addition, nucleic acids from 13 common respiratory pathogens were tested, including influenza virus, parainfluenza virus types 1–4, respiratory syncytial virus, adenovirus, coronavirus, and human parainfluenza virus. A bacterial panel was also included, comprising *Staphylococcus aureus*, *Pseudomonas aeruginosa*, *Escherichia coli*, *Salmonella typhi*, *Staphylococcus epidermidis*, and *Listeria monocytogenes*.

### Assessment of analytical reproducibility

For assessment of analytical reproducibility, DNA was extracted from five HBV standard-serum samples with concentrations ranging from 2 × 10^5^ to 2 × 10^1^ IU/mL using the method described above. Intra-assay and inter-assay reproducibility were evaluated in separate experiments. For the intra-assay reproducibility test, each gradient sample was subjected to three technical replicate VPCR amplifications within a single experimental run. For the inter-assay reproducibility test, the aforementioned gradient samples were amplified across three independent experimental runs, with three technical replicates set for each gradient in every run. The coefficients of variation (CVs) for intra-assay and inter-assay were calculated to evaluate the reproducibility of the method.

### Comparison of real-time VPCR and conventional quantitative real-time PCR (qPCR) for clinical sample detection

A total of 143 samples were randomly selected from 191 clinical specimens and quantitatively analyzed by both real-time VPCR and conventional qPCR. HBV DNA extracted from serum standards was used to generate standard curves for quantification. Using conventional qPCR as the reference standard, the diagnostic performance of the VPCR method was statistically evaluated. Specifically, the Clopper-Pearson exact binomial method was employed to assess the overall detection performance of the assay. Additionally, for the 40 samples that tested positive by both VPCR and qPCR, quantitative consistency between the methods was assessed using Pearson’s correlation and Bland–Altman analyses.

### Procedure for visual detection

The reaction composition and thermal cycling profile for endpoint fluorescence visual detection were consistent with those of the aforementioned real-time VPCR assay. Amplification can be performed either on a real-time fluorescence PCR instrument (Gentier 48E/48R, Tianlong Technology, China) or on a conventional thermal cycler (GeneTouch, Hangzhou Bioer Technology Co., Ltd., China). The endpoint amplification products generated by real-time VPCR were imaged using a UVP ChemStudio imaging system (Analytik Jena AG, Germany). For visualization, the FAM channel was excited with blue light (λ_ex_ = 494 nm, λ_em_ = 518 nm), while the VIC channel was excited with green light (λ_ex_ = 535 nm, λ_em_ = 556 nm).

## RESULTS

### Establishment and optimization of the Singleplex real-time VPCR amplification system

HBV DNA amplification was performed by conventional quantitative real-time PCR (qPCR) or real-time VPCR using the designed specific primers. After amplification for 72 and 25 min, respectively, clear and single bands were observed on agarose gel electrophoresis (Fig. S1A). The amplified products were subsequently sequenced, and the sequencing results revealed sequences identical to those deposited in GenBank (GenBank accession number: KY881949), thereby demonstrating the high specificity of the designed primers (Fig. S1B). Subsequently, the designed TaqMan probe was incorporated into the real-time VPCR system, and the amplification results were monitored using a real-time PCR instrument. The concentrations of HBV primers, probes, Mg^2+,^ and the annealing temperature were systematically optimized. As shown in Fig. S2, the VPCR assay achieved optimal amplification efficiency at a primer concentration of 1.0 μM, a probe concentration of 1.0 μM, an Mg²⁺ concentration of 6 mM, and an annealing temperature of 55°C.

Various singleplex qPCR detection systems for HBV have been reported (30, 31); however, the presence of certain components in samples or inhibitory substances in DNA extracts may result in the suppression of PCR amplification, leading to false-negative results (32). To ensure the accuracy and reliability of the detection outcomes, the human internal control gene RNAse P (33), as previously described, was incorporated as an internal control in the rapid detection system. Similarly, the optimization of the primer, probe, Mg²⁺^+^ concentration, and annealing temperature within the real-time VPCR system was conducted. The optimization results of the real-time VPCR system for the internal control gene are presented in Fig. S3A through D, where optimal amplification efficiency was achieved at a primer concentration of 0.4 μM, a probe concentration of 0.2 μM, an Mg²⁺^+^ concentration of 6 mM, and an annealing temperature of 55°C.

### Establishment of the duplex real-time VPCR system

**Fig 2:**
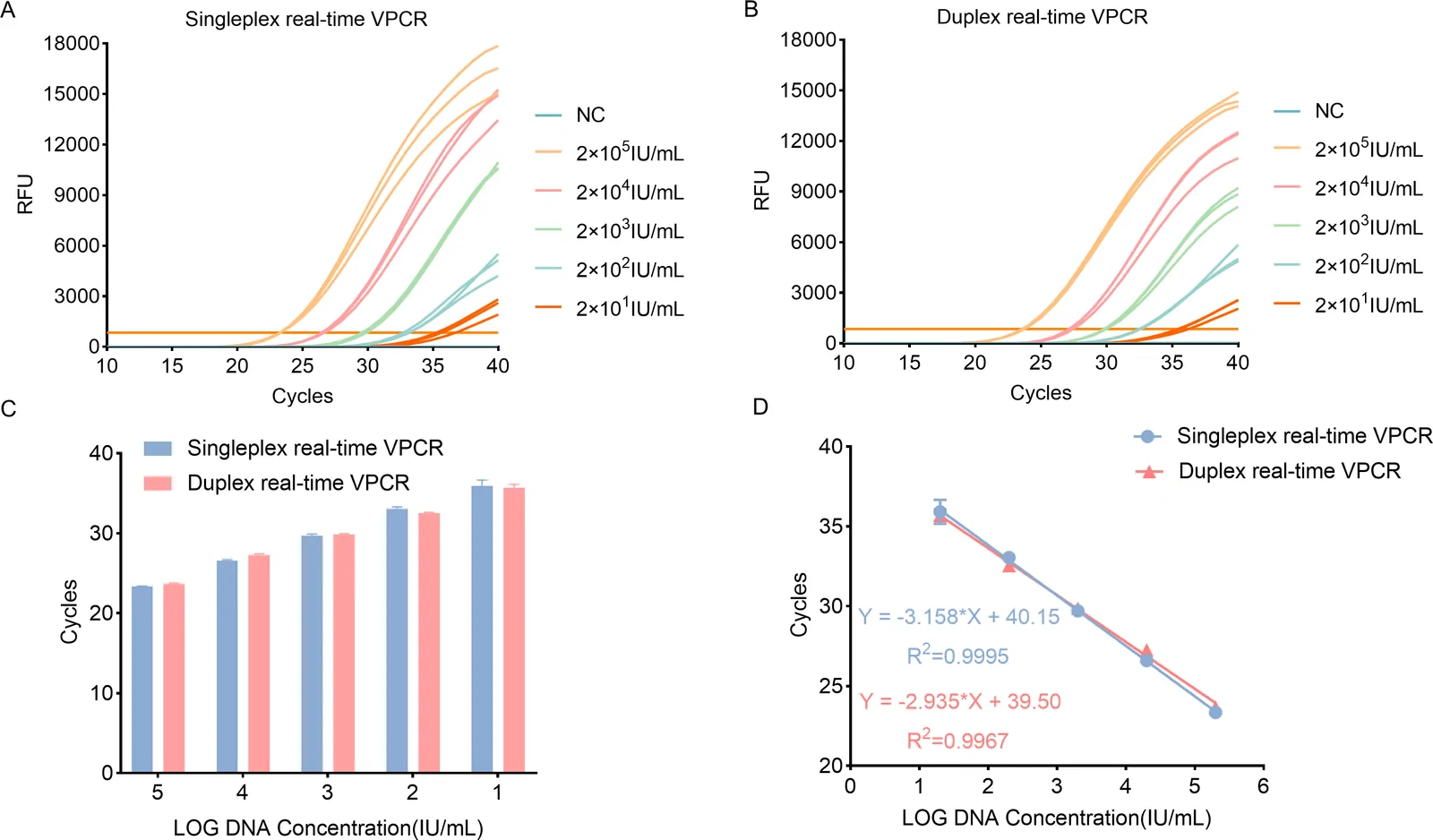
Establishment of the duplex real-time VPCR system. (**A**). Amplification curves of the singleplex real-time VPCR for detecting HBV-DNA across the concentration range of 2 × 10^5^ to 2 × 10 IU/mL. (**B**). Amplification curves of the duplex real-time VPCR for detecting HBV-DNA across the concentration range of 2 × 10^5^ to 2 × 10 IU/mL. (**C**). Ct values for HBV-DNA quantification using singleplex and duplex real-time VPCR systems. (**D**). Standard curves for HBV-DNA quantification using the singleplex and duplex real-time VPCR systems.

Considering that the optimal annealing temperature and magnesium ion concentration for the two singleplex systems were 55°C and 6 mM, respectively, these conditions were directly combined, and the primer and probe concentrations for both HBV and RNAse P were optimized to minimize interference between the two channels. As a result, a duplex real-time VPCR system was successfully developed. To evaluate its amplification efficiency, HBV DNA extracted from standard serum was used as the template. The extracted DNA was serially diluted 10-fold from 2 × 10^5^ to 2 × 10^1^ IU/mL and tested in triplicates using both the duplex and singleplex systems. The Ct values from the singleplex system served as a reference, and it was observed that the addition of the internal control did not significantly affect the amplification of HBV DNA (Fig. 2A and B; Fig. S3E), as evidenced by nearly identical Ct values (Fig. 2C) and overlapping standard curves (Fig. 2D). Thus, a duplex real-time VPCR system for HBV-DNA detection was successfully established, enabling the simultaneous detection of HBV DNA and the RNAse P internal control in a single tube while minimizing false-negative results.

### Analytical sensitivity and limit of detection

**Fig 3:**
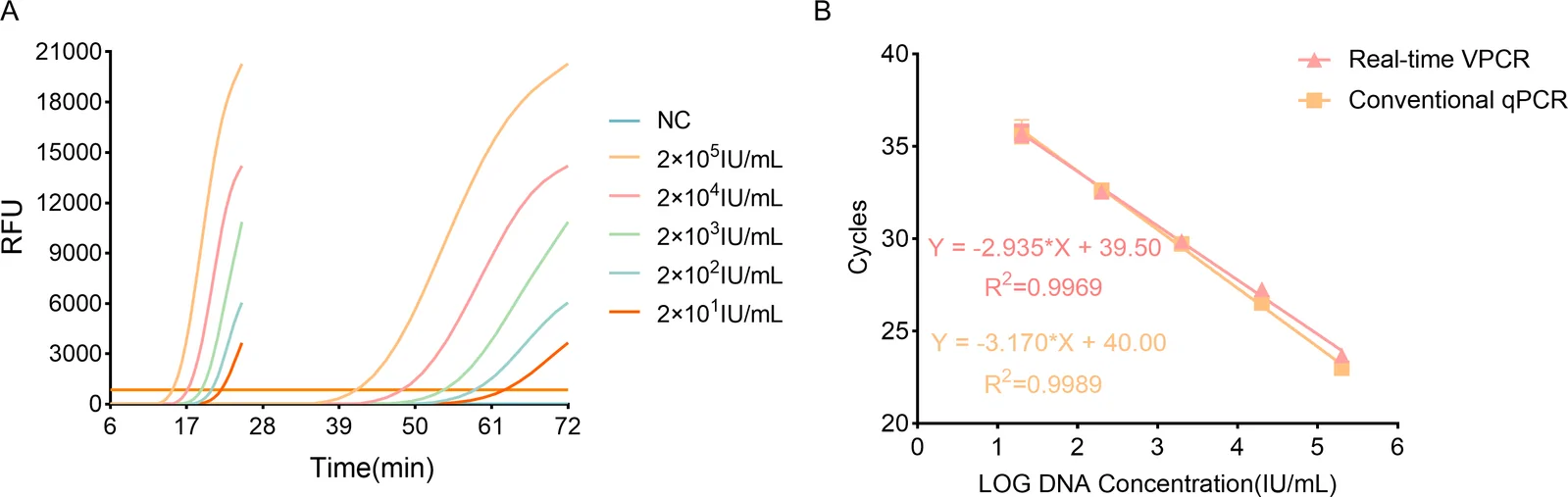
Analytical sensitivity. (**A**). Amplification curves of the developed real-time VPCR and conventional qPCR for detecting HBV-DNA extracted from HBV standard serum across a concentration range of 2 × 10^5^–2 × 10^1^ IU/ml L. (**B**). Standard curves for HBV-DNA quantification using the developed real-time VPCR and conventional qPCR systems.

Analytical sensitivity was evaluated by parallel testing of real-time VPCR and conventional quantitative real-time PCR (qPCR) using DNA extracts from HBV standard serum across a concentration range of 2 × 10^5^–2 × 10^1^ IU/ml. As shown in Fig. 3A, both methods robustly amplified HBV DNA across this range. Notably, the total amplification time was reduced from 72 min with conventional qPCR to 25 min with real-time VPCR. As indicated by the standard curves in Fig. 3B, both assays showed excellent linearity, with R^2^ values of 0.9969 and 0.9989, respectively. Moreover, the high degree of overlap between the two curves indicates that, despite the substantial reduction in amplification time, the real-time VPCR system retained the amplification efficiency of conventional qPCR. This further demonstrates the superiority of the established real-time VPCR system. Next, the limit of detection was determined by testing HBV DNA extracted from HBV standard serum at 200, 20, 15, and 10 IU/mL in 20 replicates per concentration. As shown in Table 2, HBV DNA was not reliably detected at 10 IU/mL, whereas consistent detection was achieved at 20 IU/mL. Accordingly, the LOD of the assay was defined as 20 IU/mL (Table 2).

**TABLE 2:** Limits of Detection

HBV DNA concentration IU/mL	No. of replicates	Real-time VPCR		Conventional qPCR	
		No. of detected	Detection rate%	No. of detected	Detection rate%
200	20	20	100	20	100
20	20	20	100	20	100
15	20	0	0	0	0
10	20	0	0	0	0

### Analytical specificity

**Fig 4:**
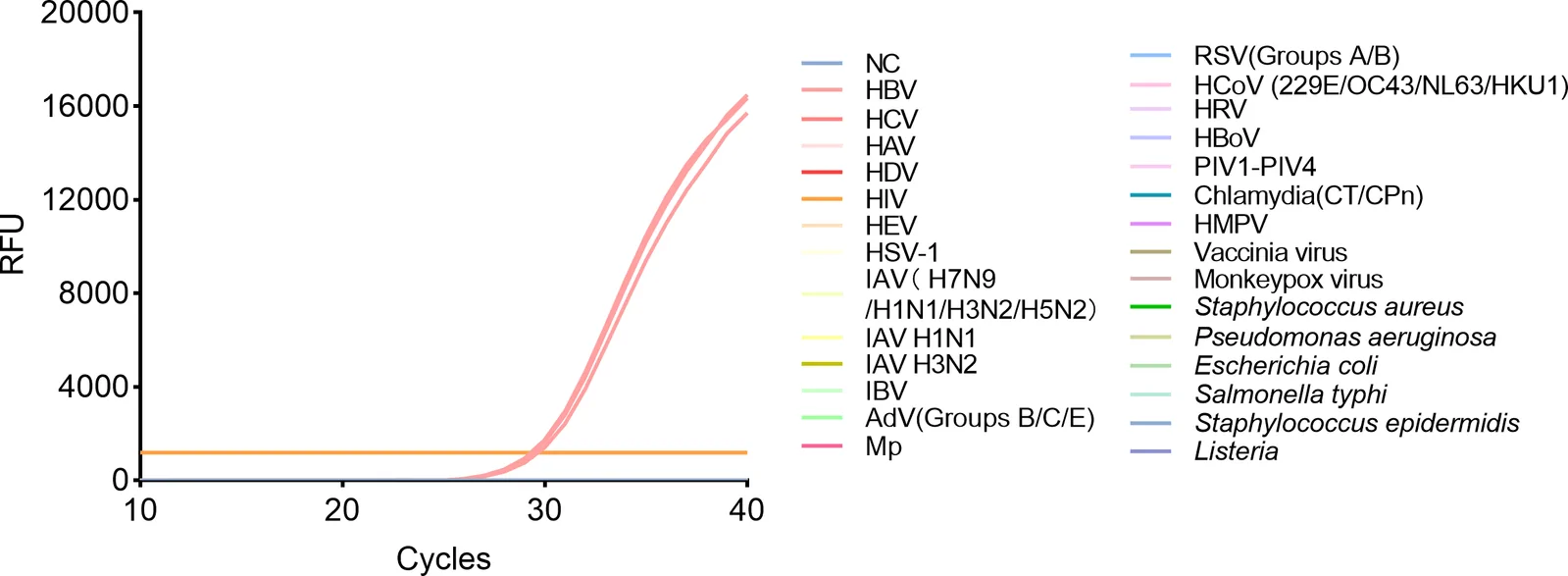
Analytical specificity of the real-time VPCR system.

To evaluate the analytical specificity of the established real-time VPCR system, the system was used to amplify the nucleic acids of vaccinia virus, monkeypox virus, *Staphylococcus aureus*, *Pseudomonas aeruginosa*, *Escherichia coli*, *Salmonella typhi*, *Staphylococcus epidermidi*s, *Listeria*, and the DNA of 13 respiratory pathogens (including IAV [H7N9, H1N1, H3N2, and H5N2), IAV-H1N1 (2009 pandemic strain), IAV-H3N2, IBV (Victoria lineage and Yamagata lineage), AdV (Groups B, C, and E), HBoV, HRV, PIV (types 1, 2, 3, and 4), HCoV (229E, OC43, NL63, and HKU1), RSV (Groups A and B), HMPV, Mp, and Chlamydia (CT/CPn)). The results showed that amplification curves were observed only for HBV, while no amplification curves were detected for the other 27 pathogens or the negative controls. This indicates that the established VPCR detection method exhibits excellent specificity (Fig. 4).

### Analytical reproducibility

Based on the established real-time VPCR system, five dilution gradients (2 × 10^5^ to 2  × 10^1^ IU/mL) of quantitative reference standards were selected for intra-assay and inter-assay VPCR amplification, and the coefficients of variation (CVs) for both experiments were calculated to evaluate the reproducibility of the method. The results demonstrated that the CVs for both reproducibility tests were consistently less than 3%, with no statistically significant differences observed. These results indicate that the method demonstrates excellent reproducibility (Table S1).

### Comparison of real-time VPCR and conventional quantitative real-time PCR (qPCR) for clinical sample detection

To evaluate the consistency of the newly established real-time VPCR method for HBV DNA detection, 143 clinical DNA samples were tested using conventional quantitative real-time PCR (qPCR) as the reference standard. Conventional qPCR identified 40 positive and 103 negative samples. In comparison, the real-time VPCR assay detected 43 positive samples, comprising 40 true positives and 100 true negatives (Table 3). The three discordant samples all exhibited relatively low viral loads, measured at 120, 40, and 33 IU/mL, respectively. Repeated analysis of these samples yielded consistent results, suggesting that real-time VPCR may possess greater sensitivity than conventional qPCR. Statistical analysis using the Clopper-Pearson exact binomial method revealed that the real-time VPCR assay achieved a sensitivity of 100% (95% CI: 91.2%–100.0%), a specificity of 97.09% (95% CI: 91.7%–99.4%), and an overall accuracy of 97.9% (95% CI: 94.1%–99.5%). These findings demonstrate that real-time VPCR offers high accuracy and consistency, making it a reliable and novel alternative to conventional qPCR for clinical HBV DNA detection.

**TABLE 3:** Comparison of diagnostic performance between real-time VPCR and conventional quantitative real-time PCR (qPCR)^*a*^

Real-time VPCR	Clinical specimens			Result
	Conventional qPCR			
	M+	M–	Total	
M+	40	3	43	Sensitivity (95%CI)=1.000(0.912-1.000)
M-	0	100	100	Specificity (95%CI)=0.970(0.917-0.994)
Total	40	103	143	Overall accuracy (95%CI)=0.979(0.941-0.995)

^*a*^ (M+) and (M–) denote positive and negative detection results, respectively.

**Fig 5:**
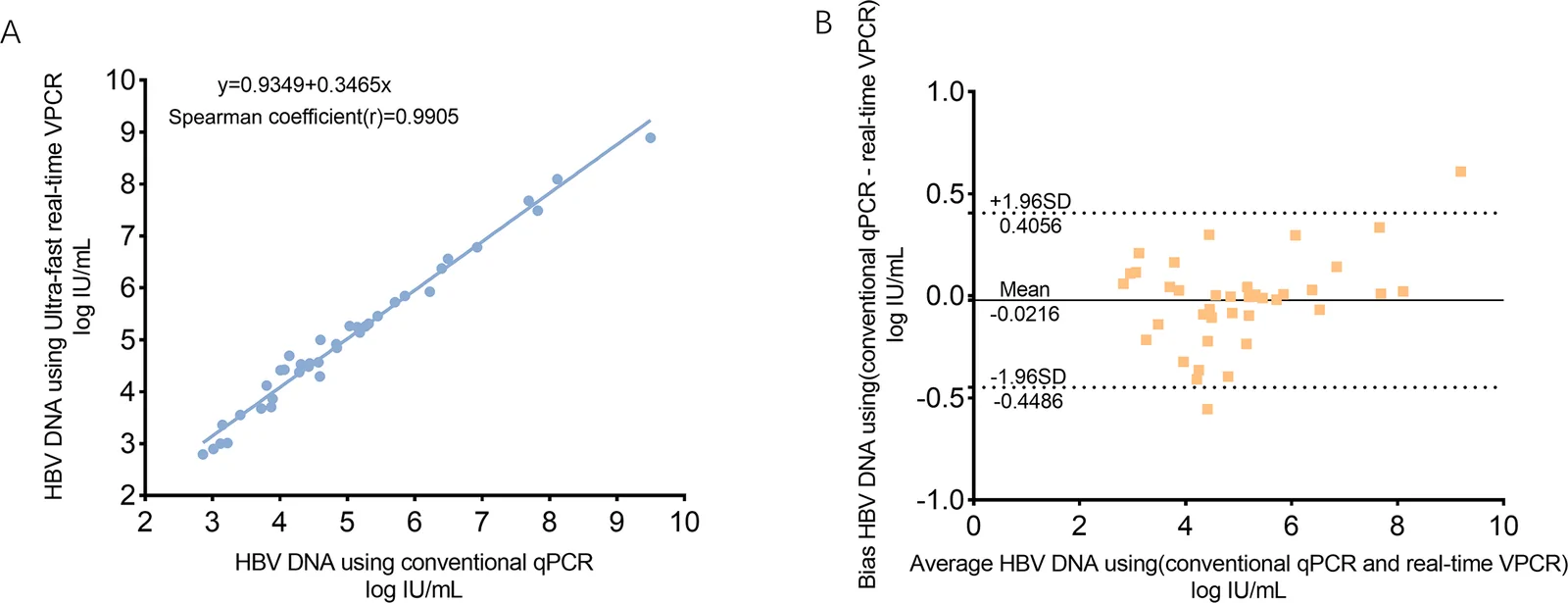
(**A**). Statistical analysis of quantitative results between real-time VPCR and conventional qPCR using Pearson regression. (**B**). Statistical analysis of quantitative results between real-time VPCR and conventional qPCR using Bland-Altman plots. Note: three discordant samples (positive by real-time VPCR but negative by conventional qPCR) were excluded from the quantitative correlation analysis, with viral loads determined by VPCR as 120, 40, and 33 IU/mL.

To evaluate the quantitative agreement between the real-time VPCR and conventional qPCR methods, the 40 samples that tested positive by both assays were analyzed using Pearson correlation and Bland–Altman analyses. First, Pearson analysis revealed a significant positive linear correlation between the two methods (r = 0.9905; 95% CI: 0.9820–0.9950), indicating highly consistent detection trends (Fig. 5A). Second, Bland-Altman analysis and paired sample *t*-tests were performed to assess potential bias (Fig. 5B; Table S2). The mean values for VPCR and conventional qPCR were 4.993 and 5.015, respectively, with a mean difference of only −0.0216. The *t*-test confirmed no statistically significant difference (*P* = 0.534), suggesting an absence of systematic bias. Furthermore, 95.0% (38/40) of the data points fell within the 95% limits of agreement (LoA). These results confirm that the developed real-time VPCR method exhibits strong quantitative agreement and consistency with conventional qPCR.

### Visual detection (including clinical samples)

**Fig 6:**
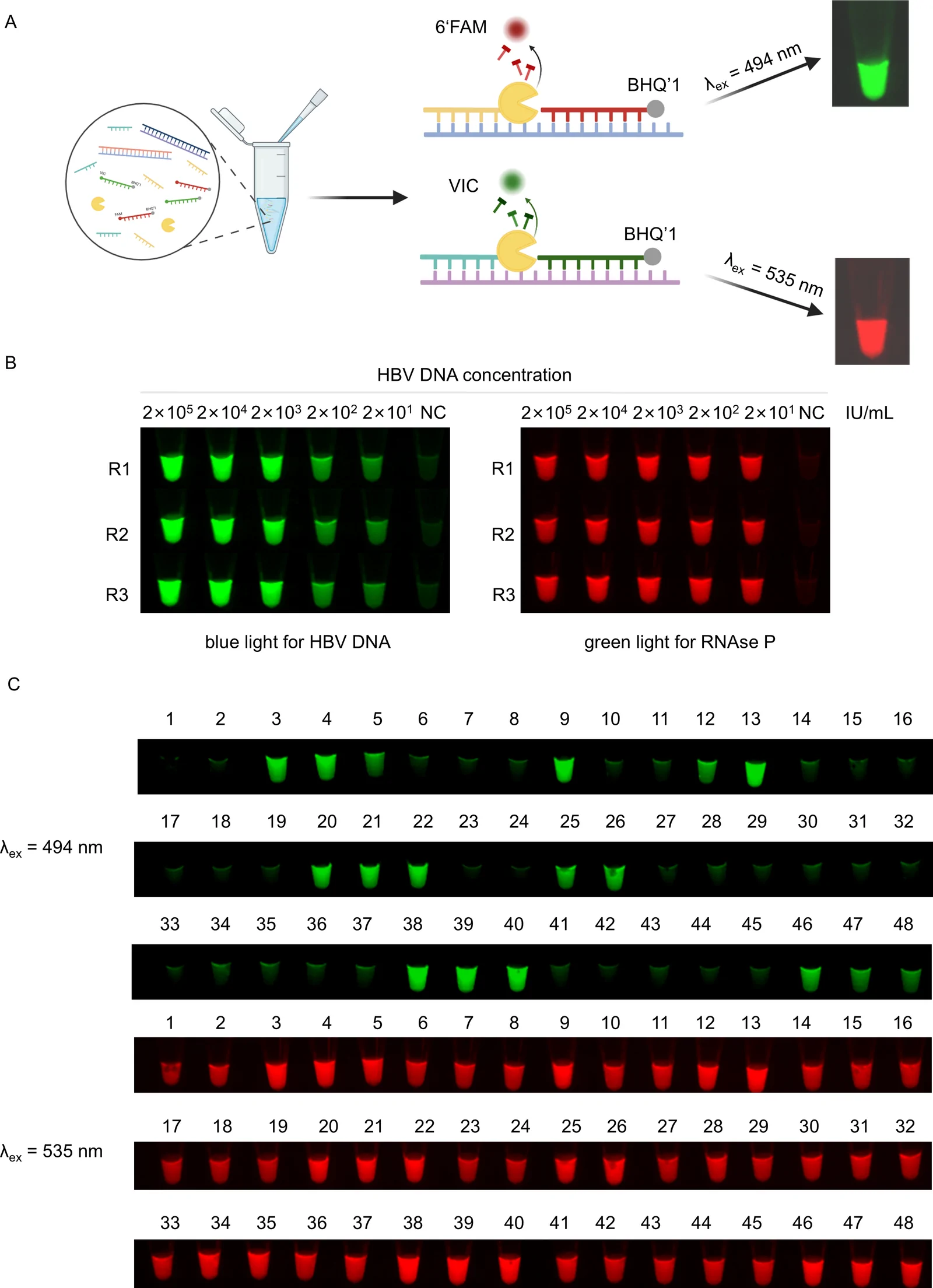
Endpoint fluorescence-based visual detection. (**A**). Schematic illustration of the probe-cleavage fluorescence mechanism under excitation light. (**B**). Endpoint fluorescence images across an HBV DNA dilution series. (**C**). Endpoint fluorescence images of 48 clinical samples.

Given that the real-time VPCR system employs TaqMan probes, in addition to real-time fluorescence monitoring, the fluorescence released upon probe cleavage can also be used for endpoint, visual readout of the results. The underlying principle is illustrated in Fig. 6A. Briefly, two TaqMan probes are incorporated into the duplex real-time VPCR assay. The HBV-specific probe is labeled at the 5′ end with 6-carboxyfluorescein (FAM), with excitation at 488 nm and maximal emission at 520 nm. The internal control probe targeting the human RNase P gene is labeled with VIC, with excitation at 535 nm and peak emission at 570 nm. Both probes carry a BHQ-1 quencher at the 3′ end. When intact, fluorescence is suppressed via fluorescence resonance energy transfer between the reporter and the quencher. During amplification, the 5′–3′ exonuclease activity of Taq DNA polymerase cleaves the hybridized probe, abolishing quenching and generating a fluorescent signal. The endpoint fluorescence is subsequently captured using an intelligent imaging system, enabling visual detection of both the HBV target and the internal control. This qualitative endpoint visualization does not require an expensive real-time quantitative PCR instrument and can be implemented with a simple, low-cost fluorescence detection device. Therefore, it provides an alternative signal readout strategy for the real-time VPCR platform and may be particularly suitable for HBV screening in resource-limited countries and regions.

Endpoint fluorescence visualization was first performed using the amplification products generated in the analytical sensitivity experiments. As shown in Fig. 6B, when HBV DNA templates were serially diluted from 2 × 10^5^ to 2 × 10^1^ IU/mL, the green fluorescence intensity in the FAM channel decreased proportionally with decreasing template concentration. Notably, even at the lowest concentration of 20 IU/mL, the fluorescence signal remained clearly detectable and could be readily distinguished from the negative control, consistent with the real-time VPCR results. In addition, the internal control RNase P signal remained stable across all dilution levels, with the VIC channel showing a consistent red fluorescence intensity. Collectively, these results indicate that the dual-color endpoint fluorescence readout enables reliable discrimination between positive and negative samples in a standard sample matrix.

To assess its applicability to clinical samples, endpoint visualization was subsequently performed on clinical specimens. From samples that had already been quantified by qPCR in routine clinical testing, 48 specimens were selected, covering high, medium, and low viral-load ranges, and the corresponding qPCR results are provided in Table S3. All specimens were tested following the developed endpoint fluorescence visualization protocol. The amplified end-products were subjected to fluorescent imaging with a UVP ChemStudio system under the respective excitation parameters for FAM and VIC channels, and the corresponding results are presented in Fig. 6C. As shown in Fig. 6C, under excitation at 494 nm, positive fluorescence signals were observed in 17 samples, namely, Tube No. 3, 4, 5, 9, 12, 13, 20, 21, 22, 25, 26, 38, 39, 40, 46, 47, and 48, and the fluorescence intensity of borderline positive samples remained distinguishable from that of negative samples. However, although Samples 8, 34, and 35 had viral loads of 30, 21, and 18 IU/mL, respectively, their fluorescence signals were not clearly distinguishable from those of negative samples. These findings indicate that endpoint visualization is suitable for rapid, large-scale screening, whereas accurate quantification for low-viral load samples still requires real-time VPCR. Under excitation at 535 nm, all samples showed consistent red fluorescence in the VIC channel, indicating successful nucleic acid extraction and amplification and suggesting the absence of false-negative results due to process failure.

## DISCUSSION

In this study, a rapid quantitative detection system was developed based on real-time fluorescence VPCR technology. By utilizing an optimized and efficient nucleic acid extraction protocol, the nucleic acid extraction step for both VPCR and conventional qPCR could be accomplished within 10 min. Among these, VPCR amplification required only 25 min, enabling the entire detection process to be completed in approximately 35 min. In comparison, conventional qPCR amplification took 72 min, resulting in a total assay time of around 82 min. Validation against the standard qPCR using 143 clinical samples demonstrated excellent performance: sensitivity reached 100% and specificity was 97.09%. Furthermore, quantitative analysis of 40 HBV-positive samples showed a strong correlation with conventional qPCR (r = 0.9905), confirming the analytical reliability and accuracy of the system. Moreover, a low-cost qualitative visual readout method utilizing probe cleavage was devised and validated in 48 clinical samples. It demonstrated robust performance in samples with medium-to-high viral loads, offering an alternative readout strategy suitable for HBV screening in resource-limited settings without requiring expensive instruments.

The clinical utility of this rapid turnaround time extends beyond convenience. Although assay speed is not the only priority for routine chronic monitoring, a turnaround time of approximately 30 min addresses three key unmet needs. First, it can support acute clinical decision-making by providing timely viral load data for triage in time-sensitive scenarios such as emergency delivery or acute liver failure. Second, it enables a test-and-treat workflow that allows diagnosis and prescription within a single visit. This is particularly valuable for patients in remote areas who must travel long distances as it avoids the need for return visits to obtain results and receive prescriptions. Finally, it improves laboratory throughput by enabling rapid batch cycling. For example, three batches can be completed in the time typically required for one standard run, thereby increasing laboratory efficiency and reducing staff workload. In summary, the developed real-time VPCR system offers a powerful solution for HBV diagnostics, balancing speed with high analytical performance. This technology not only provides a new tool for efficient clinical management and screening of hepatitis B but also serves as a valuable reference for the rapid detection of other pathogens and genetic diseases. Speed, simplicity, and cost-effectiveness are key objectives in the advancement of modern molecular diagnostic technologies. Future efforts may, therefore, focus on its integration into portable devices and automated detection platforms, thereby better meeting the practical demands of clinical application and point-of-care rapid screening.

## SUPPLEMENTAL MATERIAL

Supplemental materials jcm.00154-26-s0001.docx Supplemental experimental data, Figures S1 to S3, and Tables S1 to S3.

Graphical abstract jcm.00154-26-s0002.tif Graphical abstract.

ASM does not own the copyrights to Supplemental Material that may be linked to, or accessed through, an article. The authors have granted ASM a non-exclusive, world-wide license to publish the Supplemental Material files. Please contact the corresponding author directly for reuse.
