# Supplementary material for: Development and application of a real-time VPCR method for ultra-fast quantitative analysis and Visual Detection of HBV-DNA
Source: J Clin Microbiol. 2026 Aug 14;64(9):e00154-26. doi: 10.1128/jcm.00154-26 (PMC13556246; doi:10.1128/jcm.00154-26)
Supplement: Supplementary file 1 [file jcm.00154-26-s0001.docx]

**Development and Application of a Real-time VPCR Method for Ultra-fast Quantitative analysis and Visual Detection of HBV-DNA**

**Authors names:** Tingting Zhao^a^, Tongyi Peng^a^, Jiaqi Cui^a^, Yan Chen^a^, Wenjun Song^a^, Hongling Chen^a^, Bo Wu^b*^, Rong Chen^a**^

**Affiliations：**

a. School of pharmacy, School of Ethnic Medicine, Chinese Medicine Germplasm Resources Innovation and Effective Uses Key Laboratory of Sichuan Province, Chengdu University of Traditional Chinese Medicine, Chengdu, Sichuan, 611137, China.
b. Department of Clinical Laboratory, Hospital of Chengdu University of Traditional Chinese Medicine, Chengdu, Sichuan, 610075, China.

**Correspondence authors:**

*Bo Wu, Department of Clinical Laboratory, Hospital of Chengdu University of Traditional Chinese Medicine, No.39, Shiergiao Road, Jinniu District, Chengdu, China, 610075. E-mail addresses: duwolf108@163.com.

**Rong Chen, School of Ethnic Medicine, Chengdu University of Traditional Chinese Medicine, 1166 Liutai Avenue, Wenjiang District, Chengdu, China, 611137. E-mail addresses: [chenrong@cdutcm.edu.cn](mailto:chenrong@cdutcm.edu.cn)

Figure S1 A. Agarose gel electrophoresis results of HBV DNA amplification using VPCR and conventional PCR. M: DNA molecular weight marker (100–2000 bp); NC: negative control; PC: pCold–HBV–N standard plasmid. B. Sequencing results of HBV DNA amplification using real-time VPCR and conventional quantitative real-time PCR(qPCR).

**Figure S2.** Optimization of the ultra-fast real-time VPCR singleplex amplification system for HBV-DNA. A-D. Show the optimization results for HBV DNA primer concentration, probe concentration, magnesium ion concentration, and annealing temperature, respectively.

**Figure S3 A-D**. Show the optimization results for RNAse P reference gene primer concentration, probe concentration, magnesium ion concentration, and low temperature, respectively. E. Sensitivity experiment of the Real-time VPCR system with internal control amplification results.

**Table S1** Results of the intra-assay and inter-assay reproducibility experiment for the real-time VPCR system.

| Template | Template concentration | Intra reproductivity | | Inter reproductivity | | |
| --- | --- | --- | --- | --- | --- | --- |
|  |  | Ct (Mean ± S.D.) | CV (%) | Ct (Mean ± S.D.) | CV (%) | |
| HBV | 2×10^5^IU/mL | 23.77 ± 0.082 | 0.35 | 23.80 ± 0.145 |  | 0.61 |
|  | 2×10^4^IU/mL | 26.26 ± 0.088 | 0.34 | 26.30 ± 0.154 |  | 0.59 |
|  | 2×10^3^IU/mL | 29.38 ± 0.178 | 0.61 | 29.54 ± 0.314 |  | 1.06 |
|  | 2×10^2^IU/mL | 32.71 ± 0.105 | 0.32 | 32.79 ± 0.299 |  | 0.91 |
|  | 2×10^1^IU/mL | 36.05 ± 0.314 | 0.87 | 36.27 ± 0.235 |  | 0.65 |
| Rnase-P | 10^3^copies/μl | 32.06 ± 0.314 | 0.98 | 32.92 ± 0.403 |  | 1.22 |
|  |  | 32.59 ± 0.281 | 0.86 | 32.45 ± 0.494 |  | 1.52 |
|  |  | 31.74 ± 0.359 | 1.13 | 32.06 ± 0.672 |  | 2.10 |
|  |  | 31.77 ± 0.625 | 1.97 | 33.10 ± 0.349 |  | 1.06 |
|  |  | 32.29 ± 0.858 | 2.66 | 31.43 ± 0.567 |  | 1.80 |

| **Table S2** Descriptive Statistics and Agreement Analysis of Conventional Quantitative Real-time PCR(qPCR) vs. Real-time VPCR | |
| --- | --- |
| Item | Value |
| n | 40 |
| Mean (Conventional qPCR) | 4.993 |
| Mean (Real-time VPCR) | 5.015 |
| Mean (Difference) | -0.0216 |
| S.D. (Difference) | 0.218 |
| 95% CI (Difference Mean) | -0.091 ~ 0.048 |
| 95% CI (Difference) | -0.449 ~ 0.405 |
| t (H0: Mean Difference = 0) | -0.628 |
| p Value (H0: Mean Difference = 0) | 0.534 |
| CR (Coefficient of Repeatability) | 0.424 |

Note: n represents 40 samples that tested positive by both VPCR and qPCR methods.Original HBV DNA concentrations were log₁₀-transformed prior to analysis (unit: log IU/mL). The “Mean (Conventional qPCR)” and “Mean (Real-time VPCR)” presented in the table represent the means of log₁₀-transformed HBV DNA concentrations (unit: log IU/mL). Other statistical parameters, including the mean difference, standard deviation of the differences, 95% confidence interval, t-value, p-value, and coefficient of repeatability (CR), are dimensionless statistics and therefore have no units.

**Table S3** Detection results of 48 identified samples.

| **Sample** | HBV Load(IU/mL) | Sample | HBV Load(IU/mL) | **Sample** | HBV Load(IU/mL) |
| --- | --- | --- | --- | --- | --- |
| **1** | Not detected | **17** | Not detected | **33** | Not detected |
| **2** | Not detected | **18** | Not detected | **34** | 2.19×10^1^ |
| **3** | 2.85×10^3^ | **19** | Not detected | **35** | 1.82×10^1^ |
| **4** | 1.60×10^2^ | **20** | 2.96×10^3^ | **36** | Not detected |
| **5** | 7.26×10^2^ | **21** | 2.84×10^3^ | **37** | Not detected |
| **6** | Not detected | **22** | 3.63×10^3^ | **38** | 9.76×10^3^ |
| **7** | Not detected | **23** | Not detected | **39** | 8.94×10^3^ |
| **8** | 3.07×10^1^ | **24** | Not detected | **40** | 2.41×10^4^ |
| **9** | 2.41×10^4^ | **25** | 9.11×10^2^ | **41** | Not detected |
| **10** | Not detected | **26** | 1.19×10^3^ | **42** | Not detected |
| **11** | Not detected | **27** | Not detected | **43** | Not detected |
| **12** | 1.99×10^2^ | **28** | Not detected | **44** | Not detected |
| **13** | 1.66×10^4^ | **29** | Not detected | **45** | Not detected |
| **14** | Not detected | **30** | Not detected | **46** | 6.16×10^2^ |
| **15** | Not detected | **31** | Not detected | **47** | 2.15×10^2^ |
| **16** | Not detected | **32** | Not detected | **48** | 1.53×10^2^ |
